# DENV-4 infection suppresses transcription of DNA repair genes

**DOI:** 10.1073/pnas.2536909123

**Published:** 2026-08-04

**Authors:** Erica N. Lamkin, Jessica Reich, Josh A. Victor, Madison Guyette, Naveen Kothandaraman, Vihit Gupta, Alfred T. Harding, Tristan X. Jordan, Lee Gehrke, Pei Zhou, Benjamin tenOever, Nimrat Chatterjee

**Affiliations:** ^a^ Department of Microbiology and Molecular Genetics, University of Vermont, Burlington, VT 05405; ^b^ Institute for Medical Engineering and Science, Massachusetts Institute of Technology, Cambridge, MA 02138; ^c^ Department of Microbiology, University of Washington, Seattle, WA 98109; ^d^ Department of Laboratory Medicine and Pathology, University of Washington, Seattle, WA 98109; ^e^Department of Microbiology, Harvard Medical School, Boston, MA 021383; ^f^ Department of Biochemistry, Duke University School of Medicine, Durham, NC 27710; ^g^ Department of Microbiology, New York University Grossman School of Medicine, New York City, NY 10016; ^h^ University of Vermont Cancer Center, University of Vermont, Burlington, VT 05405

**Keywords:** DNA repair, dengue, DENV-4, translesion synthesis, genome instability

## Abstract

The molecular mechanisms behind Dengue virus-dependent host pathogenesis, especially genome instability, remain largely unclear. This RNA virus causes a debilitating disease during active infection and presents future risks of postdengue syndromes, leukemia, and DNA damage in the blood cells of infected patients, with the underlying mechanisms unknown. In this study, we show that DENV-4 infection induces significant DNA damage and suppresses the transcription of genes involved in DNA repair and select mutagenic translesion synthesis (TLS) polymerases, indicating that DENV-4-dependent pathobiology leaves durable biological “scars” that incrementally increase chronic disease risk, including carcinogenesis and postdengue syndromes.

Dengue, a significant global public health issue, causes up to 400 million cases annually, with recent increases in diagnoses in the United States (1). Without specific antivirals, limited vaccine effectiveness, and ongoing risks of postdengue syndrome, examining the pathophysiology of infected host cells is essential. The dengue virus belongs to the *Flaviviridae family* and has four serotypes (DENV-1, DENV-2, DENV-3, DENV-4), which share similar clinical symptoms and around 65% of their genomes (2). Recent epidemiological studies have linked dengue infection to an increased future risk of leukemia, with the strongest association occurring several years after the initial infection (3). Additionally, dengue infection has been shown to cause DNA damage in the blood cells of infected individuals (4), indicating that the dengue virus impacts host DNA. Interestingly, some RNA viruses, such as Hepatitis C virus (HCV), Infectious bronchitis virus (IBV), Influenza A virus, Rift Valley fever virus, Chikungunya, and severe acute respiratory syndrome coronavirus 2 (SARS-CoV-2), have been found to trigger or manipulate host genome instability (5–9). However, the extent of DENV-4 virus interaction with host DNA metabolic processes remains unclear.

## Results

**Fig. 1.**
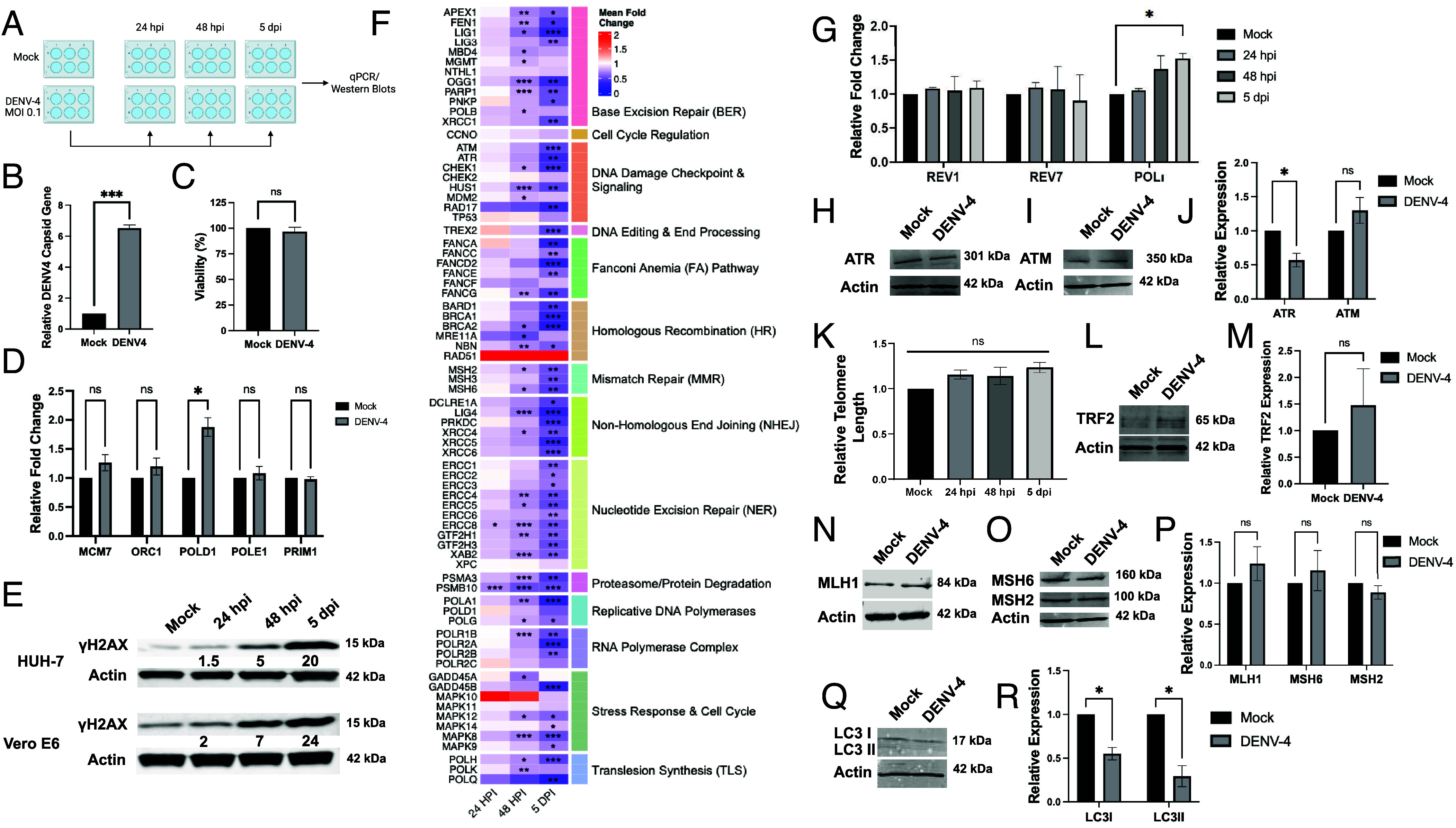
DENV-4 infection triggered DNA damage and modulated the DNA damage response in infected host cells. (*A*) Experimental timeline for DENV-4 infection, RNA isolation, qPCR testing, and western blotting. (*B*) Relative DENV-4 levels in mock versus 5 dpi (n = 3). (*C*) Relative viability of Vero E6 cells infected with DENV-4 compared to uninfected cells at 5 dpi (n = 3). (*D*) Relative expression of genes associated with replication and proliferation in mock and DENV-4-infected Vero E6 cells at 5 dpi (n = 6). (*E*) Representative western blots show time course analysis of γH2AX signaling in human HUH-7 and Vero E6 cells post-DENV-4 infection, including relative quantification levels, n = 3. (*F*) Panel shows transcript expression of base excision repair (BER), cell cycle regulation, DNA damage checkpoint and signaling, DNA editing and end processing, Fanconi anemia (FA), homologous recombination (HR), mismatch repair (MMR), nonhologous end joining (NHEJ), nucleotide excision repair (NER), proteosome/protein degradation, replicative DNA polymerases, RNA polymerases complex, stress response and cell cycle, translesion synthesis (TLS), genes. On the *Left* is a list of genes tested, with corresponding DNA repair pathway color-coded on the *Right*. Three columns show fold changes in gene expression in DENV-4-infected cells over 24 h, 48 h, and 5 dpi, with significance levels indicated by *P*-values for comparisons between infected and baseline mock conditions. A purple shade within these columns indicates the relative suppression of these genes. n = 3. (*G*) Relative fold change of expression of TLS polymerases, REV1, REV7, and POL ɩ via qPCR post-DENV-4 infection at 24 hpi, 48 hpi, and 5 dpi, n = 3. (*H*–*J*) Representative western blots show expression of ATR and ATM at 5 dpi in Vero E6 cells post-DENV-4 infection, along with their quantification levels, n = 3. (*K*) Relative telomere length quantification of mock and infected Vero E6 cells 24 h, 48 h, and 5 dpi, n = 3. (*L* and *M*) Representative western blots show expression of TRF2 at 5 dpi in Vero E6 cells post-DENV-4 infection, along with their quantification levels, n = 3. (*N*–*P*) Representative western blots show expression of MLH1, MSH6, and MSH2 at 5 dpi in Vero E6 cells post-DENV-4 infection, along with their quantification levels, n = 3. (*Q* and *R*) Representative western blots show expression of LC3II at 5 dpi in Vero E6 cells post-DENV-4 infection, including their quantification levels, n = 3. *P < 0.05, **P < 0.01, ***P < 0.001, and ****P < 0.0001 by unpaired *t* test.

In this study, we examined the impact of DENV-4, an understudied serotype, on host cells at a multiplicity of infection (MOI) of 0.1 for 5 d to monitor prolonged replication and viral dynamics. Fig. 1*A* shows the experimental timeline that allows successful infection of Vero-E6 or HUH-7 cells, yielding a high titer (Fig. 1*B*), as previously reported (10) with minimal cytopathic effect (CPE) under the microscope (11), where cell viability (Fig. 1*C*), or expression of replication genes (*MMC7, ORCI, POLE1*, and *PRIM1*) remained unchanged, while *POLD1* was significantly increased, suggesting DENV-4 infection did not impact host cell proliferation or viability (Fig. 1*D*). Next, we confirmed DNA damage in the surviving cells and found that γH2AX levels increased starting at 48 hpi and reached up to 24-fold at 5 d post infection (dpi) in surviving cells (Fig. 1*E*), suggesting that DENV-4 infection at 5 dpi causes DNA damage, including possible double-strand breaks. This effect was not observed in DENV-2-infected cells at a higher MOI for 48 h(12).

Because DENV-4 infection caused DNA damage, we hypothesized that infected cells would trigger the DNA damage response (DDR). However, qPCR analysis of a 96-well gene panel shows a surprising suppression of DDR and DNA repair genes (Fig. 1*F*). For example, genes within the base excision repair (BER), DNA damage checkpoint and signaling, DNA editing and end processing, homologous recombination (HR), mismatch repair (MMR), nonhomologous end joining (NHEJ), nucleotide excision repair (NER), proteosome/protein degradation, replicative DNA polymerases, RNA polymerases complex, stress response and cell cycle, and TLS are surprisingly suppressed, except *CCNO, CHEK2, GADD45A*, select MAP kinases, *FANCF*, p53 signaling, select polymerases, *XPC, RAD51*, and *MRE11*. DENV-2 suppressed a different pattern of DNA repair transcripts at 4 d (13). It remains to be determined why DENV-4 caused persistent suppression of DDR transcription at day 5. Similarly, qPCR confirmation of individual TLS genes revealed an upregulation of the mutagenic POL ɩ gene (Fig. 1*G*). We next confirmed DDR repression by quantifying the DDR biomarkers Ataxia-telangiectasia mutated (ATM) and Ataxia Telangiectasia and Rad3-related (ATR) and found that total ATR expression was suppressed (Fig. 1 *H*–*J*). This aligns with data in Fig. 1*F* from day 5, where *CHEK2* is not downregulated, whereas *CHEK1* is. Next, because SARS-CoV-2 induces telomere instability (7), we measured telomere lengths in DENV-4-infected cells and found no changes in telomere length (Fig. 1*K*) or TRF2 protein expression (Fig. 1 *L* and *M*), a key component of the shelterin complex that maintains telomere stability, suggesting DENV-4 infection may not impair telomeres. Since both Influenza and SARS-CoV-2 viruses disrupt mismatch repair (MMR), we tested whether MMR proteins were downregulated in infected cells. Figs. 1 *N*–*P* shows that MLH1, MSH2, and MSH6, critical MMR proteins, remain unchanged, indicating a unique and complex host response to DENV-4. Here, either DENV-4 induces reactive oxygen species (ROS) (4) or directly suppresses MMR gene transcription (14). Recent studies demonstrating that BER glycosylase activity can exacerbate replication stress during oxidative damage further support the possibility that selective suppression of DNA repair pathways may favor survival of DENV-4-infected cells (15, 16). However, the accumulation of γH2AX damage in DENV-4-infected cells might promote reliance on MMR proteins for survival (14), leading to a compensatory stability of MMR proteins, a mechanism that warrants further investigation. Finally, we hypothesized that DENV-4 might suppress DDR through autophagy induction, as observed with DENV-2 (17), and examined LC3 I and II, known autophagy markers (13). Surprisingly, we observed a decrease in LC3 I and II levels (Figs. 1 *Q* and *R*) in contrast to their upregulation during DENV-2 infections (17), suggesting a novel mechanism by which DENV-4 suppresses DNA metabolism.

## Discussion

This study shows that DENV-4 infection causes significant DNA damage in surviving host cells; however, gene expression for DNA repair (except MMR), which would repair the damage, was suppressed. We hypothesize that DENV-4 infection creates disease-vulnerability windows in infected patients, during which suppressed DNA repair leaves molecular scars that promote carcinogenesis and postdengue syndromes. Furthermore, DENV-4 exhibits unique infection biology, including stable telomeres; functional MMR, unlike SARS-CoV-2 and Influenza (7, 9); and decreased LC3 I and II expression, contrasting increased autophagy with DENV-2 (15), indicating a differential disease outcome for this virus serotype. While the spectrum of host cell responses captured here is exciting and opens avenues for exploring and expanding the network of substrates for potential vaccines and antiviral drugs, this study has notable limitations. First, the unexpected suppression of DNA repair and replication must be confirmed in infected patient specimens, including determining the cause and its consequences to aid DENV-4 replication. Despite these concerns, this study reveals critical host cell pathologies associated with DENV-4 infection, opening new avenues of research, such as profiling DNA damage across other serotypes and mining patient data to determine whether carcinogenesis following DENV is more strongly associated with a specific serotype.

## Materials and Methods

All raw data available upon request to the corresponding author. Details of Methods in *SI Appendix*.

### Cell Culturing and Infection.

Vero E6 and HUH-7 cells were infected with DENV-4 at an MOI of 0.1 and harvested at 24 h, 48 h, and 5 d postinfection. Viability of infected cells was measured at 5 dpi.

### Immunoblotting.

Mock- and DENV-4–infected Vero E6 and HUH-7 cell lysates were prepared in a Laemmli buffer containing β-mercaptoethanol and analyzed by western blotting. Membranes were probed for actin, phospho-H2AX, MLH1, LC3A/B, and TRF2, and expression signals were normalized relative to mock controls.

### RT-qPCR Analysis.

For A) select DNA repair genes, POLI, REV1, and REV7 by one-step RT-qPCR, after normalization to GAPDH, and B) 95 DNA repair genes using a TaqMan Array Human DNA Repair Mechanism plate, after reverse-transcription of RNA, and normalization to 18S rRNA of the DENV-4 infected Vero E6 cells. Relative levels calculated by ΔΔCt and significance assessed by an unpaired t-tests.

### Relative Telomere Length Quantification.

Measured in genomic DNA from DENV-4–infected Vero E6 cells by qPCR using a commercial kit with SYBR Green detection on a QuantStudio 3 system.

## Supplementary Material

Appendix 01 (PDF)

## Data Availability

All study data are included in the article and/or *SI Appendix*.
