# Supplementary material for: DENV-4 infection suppresses transcription of DNA repair genes
Source: Proc Natl Acad Sci U S A. 2026 Aug 4;123(32):e2536909123. doi: 10.1073/pnas.2536909123 (PMC13462168; doi:10.1073/pnas.2536909123)
Supplement: Supplementary file 1 [file pnas.2536909123.sapp.pdf]

Extended Methods

***In vitro* models of dengue virus infection.** Vero E6 and HUH-7 cells were chosen as cellular models for DENV-4 infection. Two models were used initially to assess DNA damage caused by DENV-4: a) Vero E6 cells, derived from the kidney of an African green monkey (Chlorocebus sabaeus, AGM), which are highly susceptible to viruses like dengue due to low interferon expression; b) HUH-7 cells, derived from male hepatocellular carcinoma, also highly susceptible to DENV infection since the liver is a primary target organ of DENV. Vero E6 cells were cultured at 37°C with 5% CO2 in DMEM (Gibco), supplemented with 10 mM non-essential amino acids (Gibco), 10 mM HEPES (Gibco), 1% penicillin-streptomycin (Gibco), and 2% fetal bovine serum (FBS, Gibco). HUH-7 cells (derived from human hepatocellular carcinoma) were grown at 37°C with 5% CO2 in DMEM (Gibco), containing 1% penicillin-streptomycin (Gibco), 1% HEPES (Gibco), and 10% FBS (Gibco).

Cells were infected with DENV-4 at an MOI of 0.1. Cell lysates were collected at 24 hours post-infection (hpi), 48 hpi, and 5 days post-infection (dpi) after examining for cytopathic effect (CPE) under a microscope. Notably, infected cells exhibited minimal CPE relative to mock controls, as previously reported (1). To evaluate cytotoxicity during DENV-4 infection, we measured relative cell viability in Vero-E6 cells at 5 dpi. Here, 100,000 Vero-E6 cells were plated per well in 6-well plates. After 5 days, the cells were trypsinized, and the cell suspension was collected. The cell suspension was counted using the TC20 Automated Cell Counter (Bio-Rad) and recorded.

Time course analysis. A time-course analysis post-DENV-4 infection of Vero E6 and HUH-7 cells was performed to determine DENV-dependent modulation of cell DNA repair and genome instability pathways. Cell lysates were collected at 24 hours post-infection (hpi), 48 hpi, and 5 days post-infection (dpi). Most viral time-course studies use 48 hpi as the endpoint due to limitations such as the virus's cytotoxicity. However, we conducted a more comprehensive analysis, including 5 dpi, allowing us to fully capture and evaluate DENV-dependent modulation of host genome instability pathways.

Immunoblotting. After infection, protein lysates from Vero E6 and HUH-7 cells were collected directly in 2X Laemmli sample buffer (Bio-Rad) and 2-mercaptoethanol (β-ME), or RIPA-based lysate collection methods per manufacturer's recommendation. Samples were separated on a Novex WedgeWell 4-20% Tris-Glycine gel (Invitrogen). Proteins were transferred onto a polyvinylidene difluoride (PVDF) membrane (Thermo Scientific) at 100V for 90 minutes using the Mini Trans-Blot Electrophoretic Transfer Cell (Bio-Rad). Membranes were blocked with either Pierce StartingBlock (PBS) Blocking Buffer (Thermo Scientific), or 5% BSA dissolved in TBS containing 0.1% Tween-20 (Fisher Scientific) for 1 hour and incubated overnight at 4°C with primary antibodies. Primary antibodies included: Actin (Invitrogen, MA1744, Mouse mAb) at 1:1000; H2AX [p Ser139] (Novus Biologicals, Edgewood, CO, USA; NB100-384, Rabbit pAb) at 1:1000; MLH1 (Cell Signaling Technology, Danvers, MA, USA; 3515S, Mouse mAb) at 1:1000; ATR (Cell Signaling Technology, Danvers, MA,

USA; 2790S, Rabbit mAb) at 1:25; ATM (ThermoFisher Scientific, 168 Third Avenue, Waltham, MA 02451, USA; C.74.4, Rabbit mAb) at 1:300; MSH2 (Abcam, 1 Kendall Sq, Bldg 200, Cambridge, MA 02139-1562; ab70270, Mouse mAb) at 1:500; MSH6 (Abcam, 1 Kendall Sq, Bldg 200, Cambridge, MA 02139-1562; ab92471, Rabbit mAb) at 1:125; LC3A/B (ThermoFisher; PA1-16931, Rabbit pAb) at 1:600; and TRF2 (Abcam, 1 Kendall Sq, Bldg 200, Cambridge, MA 02139-1562; ab108997, Rabbit mAb) at 1:1000. Membranes were washed three times for 15 minutes each with TBS (Corning) containing 0.1% Tween-20 (Sigma-Aldrich) before and after incubation with secondary antibodies for 1 hour at room temperature. Secondary antibodies included donkey anti-mouse (IRDye 680RD, Li-COR Biosciences), goat anti-mouse (IRDye 680RD, Li-COR Biosciences), or goat anti-rabbit (IRDye 800CW, Li-COR Biosciences) at 1:20,000 with 0.01% SDS and 0.1% Tween-20. Membranes were imaged with the Li-COR Odyssey CLx, and images were analyzed using Image Studio software. Bands representing the proteins of interest were normalized to actin, and DENV-infected results were further normalized to mock controls. All western blot experiments had N=3 biological replicates for statistical analysis.

RT-qPCR analysis. RT-qPCR was used to evaluate the expression of DNA repair genes following DENV-4 infection. Total RNA was extracted from Vero E6 cells after DENV-4 infection using the AllPrep DNA/RNA/Protein Kit (Qiagen). Two RT-qPCR methods were used in this study. <u>In the first method,</u> we used the SYBR Green-based iTaq universal one-step kit to probe the expression of select genes, such as POLι, REV1, REV7, POLD1, POLE, PRIM1, MCM7, and ORC1. <u>In the second method,</u> we used a TaqMan Array Human DNA Repair Mechanism panel containing 96 genes and the TaqMan Fast Advanced Master Mix to probe their expression in mocks and DENV-4-infected samples.

<u>Method 1 to quantify the expression of genes:</u>
First, RNA concentration was measured using a NanoDrop 2000 (Thermo Fisher) and diluted to 10 ng/mL with RNase/DNase-Free Molecular Grade water (Cytiva). The qPCR reaction was performed with the iTaq Universal SYBR Green One-Step Kit (Bio-Rad, #1725151). The setup included: reverse transcription for 10 minutes at 50°C, polymerase activation, and denaturation for 1 minute at 95°C, followed by 35-40 amplification cycles. Each cycle consisted of denaturation for 15 seconds at 95°C, annealing/extension, and plate reading at 60°C for 60 seconds. The housekeeping gene used for the qPCR analysis was GAPDH. Primer sequences, GAPDH Fwd 5' CTGTTGCTGTAGCCAAATTCGT 3', Rev 5' ACCCACTCCTCCACCTTTGAC 3'; POLι Fwd 5' ACAAACCGGGATTTCCTACC 3', Rev 5' TCACACTTCCTTTCCCTTGAA 3'; REV1 Fwd 5' CTTCCGCTGAGGAATTGAGAAA 3'; Rev 5' GGAGAGGAGTCGTCCAGCTT 3'; REV7 Fwd 5' TAGGGATCTGAGCCTGCGG 3', Rev 5' CTGGGTGGTGAGAGACAGG 3'; POLD1: Fwd 5' ACCTTCATCCGTATCATGGAC 3', Rev 5' ATGAAGAGTCCCGGATGTTG 3'; POLE: Fwd 5' ACGCTGGAAGAGGTGTATGGCT 3', Rev 5' GGAACGGTTCTCAGAGATGAGC 3'; PRIM1: Fwd 5' TATCGCTGGCTCAACTACGGTG 3', Rev 5' CACTCTGGTTGTTGAAGGATTGG 3'; MCM7: Fwd 5' GCCAAGTCTCAGCTCCTGTCAT 3'; Rev 5' CCTCTAAGGTCAGTTCTCCACTC 3';

ORC1: Fwd 5' CTCAAGCCTAGAACGCCACGTT 3'; Rev 5' GGAAGAGACTCAGGTACAGCAG 3'. All experiments had three biological replicates.

Method 2 to quantify the expression of genes:
Total RNA was extracted as previously described. Using the High-Capacity RNA-to-cDNA Kit (Applied Biosystems), 2000 ng of total RNA was reverse-transcribed into cDNA. Specifically, a 9 µL mixture of total RNA and water containing 2000 ng of RNA was combined with 10 µL of 2X RT Buffer Mix and 1.0 µL of RT Enzyme Mix for a total reaction volume of 20 µL. Cycling conditions were followed according to the manufacturer's instructions using the MiniAmp Plus Thermal Cycler (Applied Biosystems). A panel of 95 DNA repair genes was analyzed using the TaqMan Array Human DNA Repair Mechanism plate (Applied Biosystems). TaqMan Fast Advanced Master Mix (Applied Biosystems) was used with the following cycling program: 95°C for 20 seconds, then 40 cycles of 95°C for 1 second and 60°C for 20 seconds. 18S rRNA served as the housekeeping gene. All TaqMan-based assay experiments had three biological replicates. The following genes were tested in this panel: APEX1, ATM, ATR, BARD1, BRCA1, BRCA2, CCNO, CHEK1, CHEK2, DCLRE1A, ERCC1, ERCC2, ERCC3, ERCC4, ERCC5, ERCC6, FANCA, FANCC, FANCD2, FANCE, FANCF, FANCG, FEN1, GADD45A, GADD45B, GTF2H1, GTF2H3, HUS1, LIG1, LIG3, LIG4, MAPK10, MAPK11, MAPK12, MAPK14, MAPK8, MAPK9, MBD4, MDM2, MGMT, MRE11A, MSH2, MSH3, MSH6, NBN, NTHL1, OGG1, PARP1, PNKP, POLA1, POLB, POLD1, POLG, POLH, POLK, POLQ, POLR1B, POLR2A, POLR2B, POLR2C, PRKDC, PSMA3, RAD17, RAD51, TP53, TREX2, XAB2, XPC, XRCC1, XRCC4, XRCC5, XRCC6, ERCC8, and PSMB10.

All qPCR data were collected using the QuantStudio 3 Real Time PCR system (ThermoFisher) and analyzed using the QuantStudio Design and Analysis Software v1.5.1 using the ΔΔCT method. Statistical analysis was performed using GraphPad Prism version 9.4.1. Multiple unpaired t-tests were performed to compare the mock control to each post-infection timepoint. * $P<0.05$, ** $P< 0.01$, *** $P<0.001$, ****$P<0.0001$.

Relative Telomere Length Quantification. DNA was extracted from Vero E6 cells after DENV-4 infection using the AllPrep DNA/RNA/Protein Kit (Qiagen) and measured with a Nanodrop 2000 (ThermoFisher). The DNA was diluted to 2 ng/mL with RNase/DNase-free molecular grade H2O (Cytiva). Two primers from the Relative Telomere Length Quantification qPCR kit from ScienCell were used: one for telomeric repeats (Tel) and another for a single-copy reference on chromosome 17 (SCR). Each reaction contained 2 ng of DNA, 2 µL of primer (Tel or SCR), 10 µL of PowerTrack SYBR Green Master Mix (ThermoFisher), and enough RNase/DNase-free molecular grade water to reach a final volume of 20 µL. The PCR conditions were set to 95°C for 2 minutes, followed by 40 cycles at 95°C for 15 seconds and 60°C for 1 minute using the QuantStudio 3 Real-Time PCR system (ThermoFisher). All telomere length quantification experiments had N=3 biological replicates for statistical analysis.
Data analysis was performed with QuantStudio Design and Analysis Software v1.5.1.

1. K. D. McCormick *et al.*, Development of a robust cytopathic effect-based high-throughput screening assay to identify novel inhibitors of dengue virus. *Antimicrob Agents Chemother* **56**, 3399-3401 (2012).
